# Exploring Dietary Assessment Methods Used to Measure Individual Dietary Intakes in Low- and Middle-Income Countries and Under-Served Populations in High-Income Countries

**DOI:** 10.3390/nu17020360

**Published:** 2025-01-20

**Authors:** Janelle L. Windus, Samantha J. Stewart, Marc T. P. Adam, Connor T. Dodd, Tracy L. Burrows, Clare E. Collins, Megan E. Rollo

**Affiliations:** 1 School of Health Sciences, College of Health, Medicine and Wellbeing, University of Newcastle, Callaghan, NSW 2308, Australia; janelle.windus@uon.edu.au (J.L.W.); sam.stewart@newcastle.edu.au (S.J.S.); tracy.burrows@newcastle.edu.au (T.L.B.); clare.collins@newcastle.edu.au (C.E.C.); 2 Food and Nutrition Research Program, Hunter Medical Research Institute, New Lambton Heights, NSW 2305, Australia; marc.adam@newcastle.edu.au; 3 School of Information and Physical Sciences, College of Engineering, Science and Environment, University of Newcastle, Callaghan, NSW 2308, Australia; connor.dodd@newcastle.edu.au; 4 School of Population Health, Faculty of Health Sciences, Curtin University, Bentley, WA 6102, Australia

**Keywords:** dietary assessment methods, low-middle-income-countries, under-served populations

## Abstract

Background/Objectives: For low- and middle- income country (LMIC) settings, a global nutrition transition is rapidly emerging as diets shift, resulting in a dual burden of malnutrition. High quality dietary intake data for these populations is essential to understand dietary patterns contributing to these nutrition issues. New technology is emerging to address dietary assessment challenges; however, it is unknown how researchers conducting studies with LMIC populations or under-served groups in high-income countries adopt technology-assisted methods. This study aimed to describe the features of the dietary assessment methods used in these settings. Methods: A cross-sectional survey of members of an online international nutrition network was conducted. Participants completed an online questionnaire collecting data on dietary assessment method use, populations studied, and factors influencing method selection. Results: Of 45 participants (ranging from 27 to 60 years) who completed the questionnaire, 67% conducted dietary assessments in children aged 1–5 years, 60% in pregnant women and 60% in female adults. Dietary assessment was conducted predominantly in countries classified as LMIC (n = 50), compared to the assessment of vulnerable groups in high-income countries (n = 3). All participants reported using 24-h recalls, 80% used food frequency questionnaires, while 22% used image-based and 22% used image-assisted methods. Predominant modes were interviewer-administered, paper questionnaires and manual analysis; however, digital survey platforms were used by nearly half of participants. Conclusions: Although traditional dietary assessment methods continue to dominate in LMICs, evidence of technological-assisted method use is emerging. Technology-assisted methods, tailored to address challenges in collecting quality dietary intake data in LMICs, are becoming more accessible.

## 1. Introduction

According to the World Health Organization (WHO) [1], every country in the world exhibits one or more forms of malnutrition, from undernutrition (wasting, stunting, underweight) and micronutrient deficiencies, to overweight, obesity, and the resulting diet-related noncommunicable chronic diseases (e.g., Type 2 diabetes, cardiovascular disease and some cancers). Despite commitments to tackling malnutrition globally, progress has been unacceptably slow, and recently hindered by the Covid pandemic resulting in 150 million more people affected by hunger [2]. For low- and middle-income country (LMIC) settings, a ‘global nutrition transition’ is rapidly emerging as diets shift from traditional to ‘Western’ style with increasing processed food and beverage consumption as a result of economic progress [3]. Also impacted by declining physical activity, this nutrition transition exhibits a dual burden of undernutrition and micronutrient deficiencies, along with overweight and obesity, and non-communicable diseases [4].

Measuring what, how much and when food is consumed at the individual level is essential for understanding the nutritional adequacy of a diet and identifying suitable nutrition and priority intervention targets [5]. The lack of dietary data to support health-based policies and programs creates a barrier for achieving healthy diets [6]. High quality dietary data for a given population is essential to understand the prevalence of nutritional issues and their relationship to health outcomes, and to inform agricultural, nutrition and food safety programs and policies to address these [7], such as food-based dietary guidelines and food fortification programs [6]. Regularly surveying dietary intake is required for countries to track their progress towards their nutrition targets, while on a global level keeping them accountable to their commitments for reducing global malnutrition rates [2].

A range of dietary assessment methods can be used to collect dietary data, with varying features, strengths, and limitations including measurement errors. Retrospective methods include 24-h recalls (24R) which require participants to recall their dietary intake over the past 24 h, or food frequency questionnaires (FFQ) or diet history where participants are required to estimate their usual dietary intake over a specified period (e.g., the past 3 or 7 days, or the past month). However, data accuracy is impacted by memory recall or the ability to estimate portion size [8,9]. Prospective methods involve collecting dietary intakes in the present, including estimated food records and weighed food records; however, these tend to have a higher participant burden (e.g., weighing food) and are prone to reactivity, potentially altering participant dietary consumption [8,9]. Advances in technology have led to increased use of image-based and image-assisted methods, which use cameras (e.g., mobile phones, wearable cameras) to capture images of items being consumed. These benefit from lower participant burden, but often result in a higher analyst burden [8,10]. Other advances in dietary assessment include capturing voice recordings [11].

In LMIC settings, current methods predominantly used are 24R and FFQ, and to a lesser extent estimated or weighed food records [6]. These methods require the data collectors to be present in the field for extended periods to observe and/or directly weigh food intake, or are interview-administered 24R and therefore reliant on the memory and numeracy skills of the individuals whose diet is being measured [6]. The vast majority of LMICs collect dietary data using paper questionnaires, although electronic questionnaires are more recently being applied [6]. The challenges of collecting dietary data in LMICs include high cost, time burden, technical capacities, research infrastructure and a lack of country-specific food composition databases, resulting in their low rates of large-scale data collection [7]. Countries need to account for their specific socio-economic and cultural aspects when assessing the diets of their population, such as food supply and availability across regions, seasonality, literacy levels, food taboos and preferences [8], and therefore each country must tailor dietary assessment approaches to address their individual circumstances and needs.

A review of the inventory of individual-level dietary survey data from WHO and the Food and Agriculture Organization’s (FAO) Global Individual Food consumption data tool (GIFT) [12] identified 24R as the prominent method, used in 92% of surveys conducted in 72 LMICs [6], reflecting the most appropriate method considered for low-resource populations [8,13]. FAO produced a comprehensive resource guide specifically relevant to low-resource settings, which can assist LMICs in selecting and implementing methods for collecting dietary data [8].

A review of technology-assisted dietary assessment methods in LMICs identified that approaches that used cameras, such as mobile or smartphones, to collect intake data in the form of images showed potential for implementation in these settings, as they were not reliant on participant literacy, and images could be stored on a device not reliant on a network connection [14]. Furthermore, recent reviews of dietary surveys in LMICs on the GIFT repository [6] and assessment tools used for estimating fish and seafood intake in LMICs provide additional insight into the use of dietary assessment methods in LMICs [15]. However, a lack of information exists on the application of dietary assessment methods by researchers who conduct studies in low-income populations, and whether there is adoption of methods that are supported with technology.

This study aimed to describe the features of dietary assessment methods chosen by individuals who work within low- and middle-income countries and/or under-served populations in high-income countries.

## 2. Materials and Methods

### 2.1. Study Design

This quantitative online cross-sectional study was approved by the University of Newcastle (H-2020-0254) and Curtin University (HRE2023-0060) Human Research Ethics Committees.

### 2.2. Participant Recruitment

The survey participants were aged ≥18 years who self-identified as routinely involved in measuring the dietary intake of individuals in low- and middle-income countries (LMIC) and/or under-served groups in high-income countries as part of their role in a research group, academic institution, non-government organization (NGO) or governmental department. Their involvement was in overall project/program management (e.g., program lead/manager, research academic) or day-to-day implementation (e.g., program office, health worker, research assistant). As the survey was in the English language, participants were required to read, write and understand English.

Participants were recruited via convenience sampling through membership of international open professional network online discussion groups of the United Nations System Standing Committee on Nutrition (since the collection of data, the Committee has merged to become part of UN-Nutrition [16]. An invitation to participate was posted to all members (in an unidentifiable format) on the network’s forum list-serves ‘Ag2Nut’ (Agriculture-Nutrition Community of Practice), and ‘AREA’ (Accelerated Reduction Effort on Anaemia) including links to the participant information sheet, consent form, and the online questionnaire. Snowball sampling recruitment was also utilized as participants were encouraged to share the research information link with colleagues.

Written informed consent was obtained online prior to commencing the estimated 30-min questionnaire, which was accessible between 31 May and 5 July 2021. No remuneration was provided for participation.

### 2.3. Questionnaire

Data from participants was collected exclusively online via a self-administered questionnaire administered through the QualtricsXM system (Qualtrics, Provo, UT, USA) [17]. While three demographic questions (age, number of staff, job title) were open-ended, the majority of questions were close-coded with pre-determined single response or multiple-choice categories, and an ‘other’ option for a written text response.

Participants initially provided demographic data including their age, qualifications, and languages spoken, and details about their work including organization type, current work role, country they were based in, and number of staff supervised. Participants then answered questions on the population groups (i.e., age, sex, life-stage) for which they measure individual dietary intakes, the most common countries in which they conducted dietary assessment, their spoken languages and eating styles of the population(s) studied, the dietary assessment methods used and frequency of use, specific tools used (i.e., device or pen-paper) and source of development (i.e., internally or externally), collection mode (i.e., self- or interviewer-administered), and approaches used for data analysis and interpretation. Questions also included reasons and factors for choosing dietary assessment methods and providing feedback to study participants.

### 2.4. Data Analysis

At the end of the data collection period, data was downloaded from Qualtrics and de-identified. Univariate descriptive statistical analysis of the data was conducted using SPSS (IBM version 26), including frequencies, cross-tabulations, and mean and median calculations. Open-ended questions were analysed using simple thematic analysis. Country income classification was determined by the World Bank GNI per capita for each country [18], which was compared to the World Bank country income classifications cut-offs for low-income country, LMIC, upper-middle income country or high-income country [18].

## 3. Results

### 3.1. Characteristics of End-User Participants

The questionnaire was accessed by 108 people, with 63 commencing, 18 partially completing and 45 completing in full. Results are presented based on the 45 completed surveys, of which the median amount of time for survey completion was 26 min.

Participant characteristics are presented in Table 1. Over half of participants were female (n = 24), with a mean (SD) age of 35.2 (6.3) years. Participants were highly educated with nine out of 10 participants holding a post-graduate qualification.

Six out of 10 participants worked for a university/college, while 30% of participants worked for a government or non-government organization. Participants reported working in their current role for a mean (SD) duration of 5.2 (4.6) years, as a lecturer (24% of participants), researcher (20%), professor (16%), nutritionist (13%) or program/field manager (11%). Over half (53%) of participants reported managing staff, ranging from one to 35 staff members, with an average of 18 staff being predominantly researchers or assistants (46%), or field workers (25%).

Two-thirds (n = 34) of participant roles were based in Africa, predominantly Ethiopia (n = 17), Nigeria (n = 4), Uganda (n = 2) and Ghana (n = 2), with nine other African countries represented by a single participant each (shown in Supplementary Table S1). Eleven participants were based in one of nine Asian countries, predominantly in south or southeast Asia. Of the high-income countries, four participants worked from North America, three from Europe and two from Japan. Three participants indicated being based in three to five countries, spanning across Africa and Asia.

Other than English, languages spoken by participants included Amharic (Ethiopian language) (n = 9) and other African languages (n = 8) including Afan, Afo, Chiyao and Sidamu. Other reported languages were Spanish (n = 4), French (n = 3), Italian (n = 2), Greek (n = 2), Hindi (n = 2) and Portuguese (n = 2). Japanese, Fijian, Albanian and Vietnamese were each spoken by one participant.

### 3.2. Dietary Assessment Populations and Practices

Two-thirds (67%) of dietary assessments were conducted in population samples of young children aged 13–59 months, 60% with pregnant women and 60% with female adults (Table 2, Column 1). Differences in population groups were evident between countries, with African countries conducting more studies among breastfeeding women (61% African vs. 50% non-African), while non-African countries predominantly collecting data from children 13–59 months old (86% non-African vs. 58% African).

Dietary assessments were conducted across a wide range of frequencies, from weekly to two-to-four times a year, although every one-to-three months was the prominent frequency for most population groups (Supplementary Table S2).

Africa represented the majority (87%) of countries where dietary assessments took place, most prominently Ethiopia (n = 20), followed by Asian countries with 29% participants (Supplementary Table S1). Overall, 46% of countries where dietary assessments were conducted were low-income countries, 34% were low-middle-income countries, 15% upper-middle income countries (UMIC) and 5% were high-income countries.

English was the predominant language spoken by 58% of the population groups being studied. The Ethiopian language Amharic was spoken by 13% of study populations, while 18% spoke one of 20 tribal languages mentioned, including Afan, Bengali, Chichewa, Chiyao, Malayalam and Sidamese. Tamil, French, Spanish and Hindi languages together were spoken by 22% of study populations.

### 3.3. Factors Impacting on Selection of Dietary Assessment Methods

Describing dietary intake was the reason given by the majority (80%) of participants for measuring individual dietary intake; however, examining associations between dietary intakes and dependent (64%) or independent (69%) variables were also common reasons. Health programs and/or interventions also prompted the measurement of dietary intakes for two-thirds of participants. Other reasons suggested by participants were tracking dietary pattern changes, measuring the impact of COVID-19 or as a validation indicator. No differences in reasons for conducting dietary assessment were evident between African and non-African countries.

Participant characteristics (76%) and participant burden (67%) were key considerations impacting the dietary assessment method selected by participants in this study. Factors in administering the study also influenced the dietary assessment method used, particularly cost (76%), but also time (67%), data provided (64%) and fieldworker burden (62%). Cost impacted the dietary assessment method used for most participants working in African countries (80% African vs. 64% non-African). Participants also indicated cultural dietary practices, and endorsements by international standards of assessment or standard operating procedures impacted their choice of method.

Participants reported on the usual eating styles for each population(s) studied, with similar proportions of discrete (i.e., own plate) serves only (36%) and shared (shared meal between two or more people from a single vessel) serves only (38%), while about half of the populations (58%) served meals as a combination of discrete and shared serves. Shared meals were considerably more common in African (52%) countries than non-African (7%) countries, and a combination of discrete and shared meals were more common in non-African countries (48% African vs. 79% non-African).

### 3.4. Dietary Assessment Methods Used

**Figure 1:**
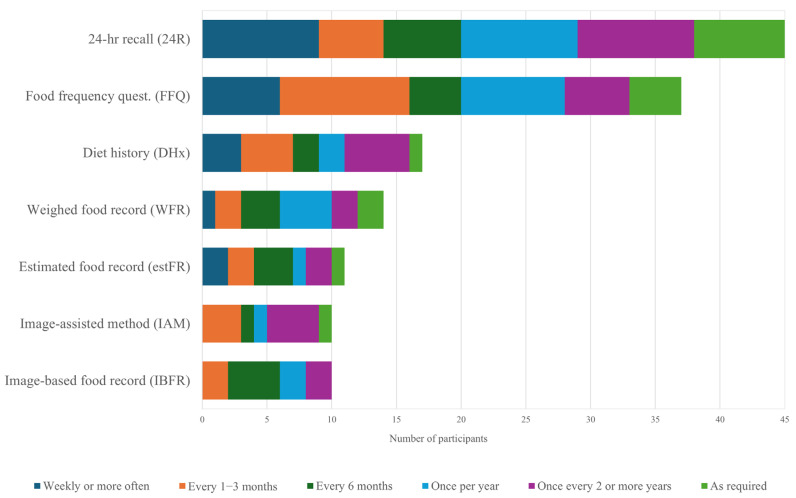
Frequency of dietary assessment methods used by participants.

All participants reported using the 24R method, with 20% indicating that they used it weekly or more often (Figure 1). Four in five (80%) participants used FFQs, of which nearly half (43%) used it at least every 3 months. The use of food record methods was reported as 38% using diet history (DHx), 31% using weighed food records (WFR) and 24% using estimated food records (estFR). Methods utilizing images were less common, with one in five (22%) participants indicated that they used image-based and 22% that they used image-assisted methods. Participants who used dietary assessment methods with less regularity or ad-hoc recorded them ‘as required’. Other methods that participants reported using included diet quality tools or dietary diversity score questionnaires. Supplementary Table S2 reports the frequency of each dietary assessment method used.

The dietary assessment methods applied to population groups are shown in Table 2. Overall, the 24R method was the most prominent approach used over all other methods for every population group, particularly women, infants and young children. FFQ was the second-most used method, particularly in studies with adult women. Diet history (DHx), weighed food record (WFR) and estimated food record (estFR) methods were used to a lesser extent, with similar proportions evident across each population group. The use of image-based and image-assisted methods was lower, yet similar across all population groups.

### 3.5. Approaches to Data Collection, Analysis and Interpretation in Dietary Assessment

Data collection predominantly used the interviewer-administered mode for all dietary assessment methods; eight in ten (82%) participants used interviewers to collect 24R, just over half (56%) used a FFQ and 24% collected DHx (Supplementary Table S3). Comparatively, a self-administered mode was less commonly used, applied by 18% of participants using a FFQ and 16% using 24R. Participants working in African countries tended to use self-administered mode more than those working in non-African countries across all dietary assessment methods; however, interview-administered remained the prominent mode for data collection for Africa.

In this study, participants predominantly reported the use of pen-and-paper records to collect dietary intake across all dietary assessment methods, specifically 24R (69%) and FFQ (49%), and at a notably higher rate in African countries (Supplementary Table S4). Digital survey platforms were being used to collect 24R (reported by 44% of participants) and FFQ (31%), while technological devices such as stand-alone mobile apps or wearable devices are emerging as collection methods, but are currently used by few participants in this context.

In terms of the development of tools used, participants reported the majority were developed internally (n = 66), specifically half (51%) of tools used for 24R and one-third (36%) of FFQ tools. Of those who used image-assisted tools, nearly all (88%) were developed internally, whereas generally each food record method was similar in being developed internally or externally.

Participants predominantly reported conducting analyses manually for most dietary assessment methods, where the analyst used either generic software such as Microsoft Access or Excel, or a specialized standalone or purpose-built nutrition assessment software (Supplementary Table S5). Most of the data collected via 24R (76%) or FFQ (53%) were manually analysed. Semi-automated analysis, i.e., manual analysis supported by some automated tasks, was used by about one-quarter of participants for 24R (29%), FFQ (24%) or WFR (16%). Fully automated analysis, i.e., no analyst involvement, was less common, with only 11% of 24R being analysed using this approach.

Almost all participants (93%) indicated comparing dietary intake data to nutrient intake recommendations or food guidance systems. For this type of dietary data interpretation, participants used semi-automated approaches (i.e., analyst used one or more automated interpretation tasks) for nearly all dietary assessment methods more commonly than manual or fully-automated (i.e., no analyst involvement) approaches (Supplementary Table S6). Over half of participants (51%) used semi-automated interpretation for 24R, 42% for FFQ and 22% for DHx. Manual interpretation approaches were used by 38% of participants for 24R, 24% for FFQ and 11% for each food record method (WFR, estFR and DHx). Fully-automated approaches were used by 7% of participants for both 24R and FFQ, 4% for WFR and 2% for estFR.

Half of participants (49%) indicated that they provided feedback to dietary assessment respondents. Feedback included diet improvement suggestions (36%) or nutrient intake adequacy summaries (31%), usually provided in verbal (42%) or physical form (24%) rather than digital (4%), while three participants indicated that they presented feedback in community education sessions or displayed communication materials in the village.

## 4. Discussion

The focus of the current study was to describe the features of dietary assessment methods used by members of an international nutrition network discussion group who self-identified as regularly measuring dietary intakes in LMICs and under-served populations in high-income countries. Participants overall were highly educated, with the majority working with African populations, particularly in Ethiopia. The participant characteristics of the current study are likely reflective of the membership characteristics of the network and/or their interest in participating in the study. However, while our study did not aim to represent dietary assessment workers in LMICs, a high representation of African countries was evident in a systematic review of dietary surveys in LMICs across a four-decade period [6].

The dominance of dietary assessment studies conducted amongst population samples of women and/or young children under five years of age was evident in this study, and is not surprising given the strong focus on the nutritional situation of these population groups by influential global health organizations such as WHO, FAO and the United Nations’ Sustainable Development Goals over the past two decades in LMICs [19]. The higher nutrient needs of women in their reproductive years, infants and young children from LMICs or under-served populations puts them at heightened risk of malnutrition or micronutrient deficiencies, necessitating regular monitoring of their dietary intake. Women of reproductive age were the most common population group in the dietary surveys conducted in LMICs over the past four decades, followed by children under two years of age [6].

The general global popularity of the 24R dietary assessment method [6] was reflected in the survey results through its universal use by all participants. The FFQ was also popular, used by four in five participants in this study, being a tool that has predominantly been used for measuring dietary intakes of specific food items or nutrient sources [15]. The 24R has been recognized as a preferred method by LMICs due to their standardized approach with local interviewers conducting data collection, thus overcoming any low literacy barriers, whereas the standard FFQ requires substantial language and cultural adaptation and validation before being implemented [7].

The current study identified cost and time as prominent considerations impacting the dietary assessment method chosen by these participants working in low-income populations, which have been found as critical barriers to conducting dietary assessment facing LMICs generally [7,14]. The inefficiencies of collecting and analysing dietary data are heightened by a weak research infrastructure in LMICs due to the high cost in establishing the resources and capacity required for dietary assessment, such as food composition databases [20], automated data collection platforms [7,14] in local languages, and nutrition-trained fieldworkers [8,21]. However, there are quality dietary data collection resources designed for low literacy populations available for countries with low resources, such as INDDEX24 [22] which has been shown to be a cost-effective method for collecting dietary intakes in LMICs [23]. Quality dietary data that is available and accessible is a prerequisite for developing evidence-based programs and policies aiming to achieve healthy diets for populations [6]. Making dietary intake data available for researchers and policymakers to use for a given population would also address cost and time barriers for many LMICs, such as FAO/WHO’s global individual food consumption data tool (GIFT) [12]. This solves a barrier that many LMICs experience with a lack of a centralised location for storing dietary data [7].

The lower prevalence of self-administered methods reported in this study may be a reflection of the challenges that can arise when using these types of tools in the LMIC setting due to variable literacy levels [21]. While interview-administered methods generally overcome literacy barriers, they are the most costly due to the high reliance on needing a local trained enumerator to collect dietary data [23]. Some use of image-based or image-assisted methods was evident in this current study, which are methods that have potential for reducing collection costs due to lower reliance on literacy skills in the self-collection of dietary data [10]. Using image-based methods is an emerging area with few studies conducted in LMICs to date, although studies such as the VISIDA image-voice-based system developed by our team [24] have demonstrated potential in Cambodia [25] and Tanzania [26], with further research required.

Recent initiatives have focused on improving access to technology-assisted dietary assessment tools in LMICs, with these approaches designed to address the barriers and challenges presented in these settings. For example, Intake–Centre for Dietary Assessment [27] provides toolkits and technology for measuring diet quality. In addition, INDDEX24 is a comprehensive online 24R platform that uses a web application and database for collecting individual dietary data [22]. INDDEX24 is user-friendly, context-adaptable and available at low or no cost for LMICs [22]. Another example is our VISIDA system which uses a self-administered image-voice food record captured via a custom smartphone application as the primary tool for the collection of individual level dietary intake data, with this data processed in a semi-automated manner in a web platform by a trained analyst [24]. Our team is currently working towards making this system available for other groups and organizations to use [28]. Finally, hosted by the FAO, GIFT is a global repository of individual food consumption data that is easily accessible online for policymakers and researchers in any country, including LMICs, to use for informing nutritional programs and interventions based on quality evidence-based data to improve nutritional status [12,29]. It is imperative that the benefits of these resources are promoted to nutrition researchers and policymakers in LMICs for their consideration, and to support regular monitoring and surveillance of dietary intakes of priority population groups.

As well as promoting the use of these above-mentioned resources in LMICs, other practical suggestions from this study include developing collaborative relationships between national researchers in LMICs and experienced international researchers, to create opportunities to co-design research projects. This would enhance understanding and skills in both national and international researchers for applying dietary assessment research in LMIC settings, while providing training and support where required. Further research into identifying facilitators and barriers of dietary assessment in each LMIC context is also recommended to more appropriately apply dietary assessment approaches, with a focus on using technological methods.

The current study aimed to describe dietary assessment features from a convenience sample of members of an international nutrition network discussion group, and this is acknowledged as a study limitation. However, comparisons with a larger, robust systematic review of dietary assessment methods used in LMICs reflected several consistent data outputs, such as population groups and methods and modes used [6]. In addition, the number of participants who assessed dietary intake in vulnerable and/or under-served groups in high-income countries was low, and is likely reflective of the online nutrition network’s objectives, which in turn would impact membership. Another limitation is that the survey was in English, which is likely to have limited participation from some individuals given the LMIC focus. Finally, given the small sample size of this study, the findings cannot be generalized to the methods used to assess individual level dietary intakes in all LMIC contexts.

## 5. Conclusions

In a small survey of participants who predominantly assess dietary intake in LMICs, the majority conducted measures of intake in young children, pregnant women and female adults. All respondents reported using 24R, with just over one-fifth reporting the use of more recent imaging methods to assess individual level intake. Most methods required an interviewer to facilitate collection, with few self-administered methods used in LMICs. Use of technology-assisted approaches is evolving, with close to half of participants indicating that they used an online survey tool to support data collection. Recent advances have seen the development of examples of technology-assisted dietary assessment methods designed for LMIC settings, with efforts concentrated on ensuring accessibility. Ongoing investment is needed to support LMICs in implementing and further refining technology-assisted dietary assessment methods.

## Figures and Tables

**Table 1:** Characteristics of participants, including demographics, country, education and employment details.

Participant Demographics, Education, Language and Country (N = 45)		n (%)
Gender	Female	24 (53.3)
	Male	18 (40.0)
	Non-binary/self-described	2 (4.4)
	Prefer not to say	1 (2.2)
Age groups	18–29 years	5 (11.1)
	30–39 years	28 (62.2)
	40–49 years	11 (24.4)
	≥50 years	1 (2.2)
Education level attained	Diploma	1 (2.2)
	Bachelor	3 (6.7)
	Post-graduate	41 (91.1)
Organization type of current job role	University/college	27 (60.0)
	Government	7 (15.6)
	Non-government	7 (15.6)
	Other: consultancy, research institute, UN	4 (8.9)
Years in current job role ^1^	<1 year	7 (15.5)
	1 to <3 years	9 (20.0)
	3 to <5 years	9 (20.0)
	5 to <7 years	6 (13.3)
	7 to <10 years	9 (20.0)
	≥10 years	4 (8.9)

^1^ 1 missing value.

**Table 2:** Population groups and continents where dietary assessments were conducted by total participants (N = 45), and by dietary assessment method.

Dietary Assessment Studies Conducted by…	Total (Multiple Responses)	Dietary Assessment Methods Used by Population Groups							
		24-h Recall (24R)	Food Frequency Quest (FFQ)	Diet History (DHx)	Weighed Food Record (WFR)	Estimated Food Record (estFR)	Image-Based Food Record (IBFR)	Image-Assisted Method (IAM)	Other ^1^
Population Group	n (%)	n (%)	n (%)	n (%)	n (%)	n (%)	n (%)	n (%)	n (%)
Infants 0–12 months	24 (53.3)	21 (46.7)	11 (24.4)	6 (13.3)	7 (15.6)	6 (13.3)	4 (8.9)	5 (11.1)	2 (4.4)
Children 13–59 months	30 (66.7)	28 (62.2)	13 (28.9)	8 (17.8)	6 (13.3)	6 (13.3)	4 (8.9)	5 (11.1)	3 (6.7)
Children 5–9 years	14 (31.1)	16 (35.6)	11 (24.4)	8 (17.8)	6 (13.3)	5 (11.1)	5 (11.1)	3 (6.7)	2 (4.4)
Adolescents 10–19 years	19 (42.2)	21 (46.7)	13 (28.9)	9 (20.0)	6 (13.3)	4 (8.9)	3 (6.7)	3 (6.7)	4 (8.9)
Pregnant women	27 (60.0)	23 (51.1)	15 (33.3)	7 (15.6)	9 (20)	8 (17.8)	5 (11.1)	5 (11.1)	3 (6.7)
Breastfeeding women	26 (57.8)	24 (53.3)	14 (31.1)	7 (15.6)	7 (15.6)	7 (15.6)	4 (8.9)	5 (11.1)	3 (6.7)
Adult females ≥20 years	27 (60.0)	27 (60.0)	18 (40.0)	6 (13.3)	8 (17.8)	6 (13.3)	4 (8.9)	6 (13.3)	5 (11.1)
Adult males ≥20 years	16 (35.6)	19 (42.2)	13 (28.9)	6 (13.3)	4 (8.9)	5 (11.1)	4 (8.9)	5 (11.1)	2 (4.4)
Other populations ^2^	6 (13.3)	7 (15.6)	4 (8.9)	3 (6.7)	3 (6.7)	3 (6.7)	1 (2.2)	3 (6.7)	0 (0)
Continent									
Africa	39 (86.7)	28 (62.2)	24 (53.3)	11 (24.4)	9 (20)	7 (15.6)	6 (13.3)	4 (8.9)	6 (13.3)
Asia	13 (28.9)	11 (24.4)	8 (17.8)	2 (4.4)	3 (6.7)	1 (2.2)	1 (2.2)	2 (4.4)	5 (11.1)
Europe	4 (8.9)	2 (4.4)	0 (0.0)	0 (0.0)	0 (0.0)	0 (0.0)	0 (0.0)	0 (0.0)	0 (0.0)
Oceania	3 (6.7)	2 (4.4)	1 (2.2)	1 (2.2)	0 (0.0)	1 (2.2)	1 (2.2)	2 (4.4)	0 (0.0)
South America	2 (4.4)	2 (4.4)	0 (0.0)	0 (0.0)	0 (0.0)	0 (0.0)	0 (0.0)	0 (0.0)	0 (0.0)
North America	1 (2.2)	1 (2.2)	1 (2.2)	0 (0.0)	0 (0.0)	0 (0.0)	0 (0.0)	0 (0.0)	0 (0.0)

^1^ Other methods mentioned by participants: 7-day FFQ of targeted items, Diet quality tool, Dietary Diversity Score for Women, Food Atlas, Verbal interview format, Dietary Diversity Score for IYCF, duplicate method; ^2^ Other populations mentioned by participants: 0–24 months, adults > 15 years, children 6–23.9 months, households (not further defined).

## Data Availability

The raw data supporting the conclusions of this article may be made available by the authors on request.

## Supplementary Materials

The following supporting information can be downloaded at: https://www.mdpi.com/article/10.3390/nu17020360/s1, Supplementary File S1 presents tables of responses to survey questions on features and approaches to dietary assessment methods used to measure individual dietary intakes that are not reported in this paper.
