# Supplementary material for: Exploring Dietary Assessment Methods Used to Measure Individual Dietary Intakes in Low- and Middle-Income Countries and Under-Served Populations in High-Income Countries
Source: Nutrients. 2025 Jan 20;17(2):360. doi: 10.3390/nu17020360 (PMC11769385; doi:10.3390/nu17020360)
Supplement: Supplementary file 1 [file nutrients-17-00360-s001.zip › nutrients-3425655-supplementary.pdf]

Supplementary File S1: Participant responses to survey questions on features and approaches of dietary assessment methods used to measure individual dietary intake

Supplementary Table S1: In which country/ies are you based in your current job role, and do you most commonly undertake dietary assessment?

Continent	Countries	Income Classification	Location based for job role		Location where dietary assessment conducted	
			n	(%)	n	(%)
Africa	Angola	LMIC	1	2.2%	1	2.2%
	Burkina Faso	LIC	1	2.2%	2	4.4%
	Cameroon	LMIC	1	2.2%	1	2.2%
	Chad	LIC	1	2.2%	0	0.0%
	Dem.Rep. of Congo	LIC	1	2.2%	0	0.0%
	Eritrea	LIC	1	2.2%	0	0.0%
	Ethiopia	LIC	17	37.8%	20	44.4%
	Ghana	UMIC	2	4.4%	3	6.7%
	Kenya	LMIC	1	2.2%	0	0.0%
	Malawi	LIC	0	0.0%	2	4.4%
	Mozambique	LIC	0	0.0%	1	2.2%
	Nigeria	LMIC	4	8.9%	4	8.9%
	Senegal	LMIC	1	2.2%	0	0.0%
	Somalia	LIC	1	2.2%	1	2.2%
	Uganda	LIC	2	4.4%	2	4.4%
	Zambia	LMIC	0	0.0%	2	4.4%
Asia	Bangladesh	LMIC	0	0.0%	1	2.2%
	India	LMIC	2	4.4%	3	6.7%
	Indonesia	UMIC	1	2.2%	1	2.2%
	Japan	HIC	2	4.4%	0	0.0%
	Lao PDR	LMIC	1	2.2%	1	2.2%
	Myanmar	LMIC	1	2.2%	1	2.2%
	Nepal	LMIC	1	2.2%	0	0.0%
	Sri Lanka	LMIC	1	2.2%	2	4.4%
	Timor-Leste	LMIC	1	2.2%	1	2.2%
	Vietnam	LMIC	1	2.2%	2	4.4%
	Yemen	LMIC	0	0.0%	1	2.2%
Oceanic	Fiji	UMIC	2	4.4%	2	4.4%
	Solomon Islands	LMIC	0	0.0%	1	2.2%
South America	Ecuador	UMIC	0	0.0%	1	2.2%
	Guatemala	UMIC	1	2.2%	1	2.2%
North America	United States	HIC	4	8.9%	1	2.2%
Europe	Albania	LMIC	0	0.0%	1	2.2%
	Hungary	HIC	1	2.2%	1	2.2%
	Italy	HIC	1	2.2%	1	2.2%
	United Kingdom	HIC	1	2.2%	0	0.0%

total	*55*	122.2%	*61*	135.6%
LIC	24	43.6%	28	45.9%
LMIC	18	32.7%	22	36.1%
UMIC	4	7.3%	8	13.1%
HIC	9	16.4%	3	4.9%

LIC, low-income countries; LMIC, low-middle-income countries UMIC, upper-middle income countries; HIC, high income countries. Participants could provide multiple responses to this question.

Supplementary Table S2: For the dietary assessment method(s) you indicated that you currently use, how frequently do you use these methods to measure individual dietary intakes within your population group(s)?

	**Infants 0-12 months**	**Children 13-59 months**	**Children 5-9 years**	**Adolescents 10-19 years**	**Pregnant women**	**Breastfeeding women**	**Adult females >20 years**	**Adult males >20 years**
	n (%)	n (%)	n (%)	n (%)	n (%)	n (%)	n (%)	n (%)
Daily	0 (0.0)	1 (2.2)	0 (0.0)	1 (2.2)	2 (4.4)	3 (6.7)	1 (2.2)	1 (2.2)
Weekly	3 (6.7)	3 (6.7)	4 (8.9)	3 (6.7)	4 (8.9)	2 (4.4)	3 (6.7)	2 (4.4)
Every 1-3 months	5 (11.1)	5 (11.1)	4 (8.9)	5 (11.1)	5 (11.1)	6 (13.3)	5 (11.1)	4 (8.9)
Every 6 months	4 (8.9)	4 (8.9)	2 (4.4)	0 (0.0)	2 (4.4)	3 (6.7)	3 (6.7)	0 (0.0)
Once per year	3 (6.7)	5 (11.1)	0 (0.0)	2 (4.4)	1 (2.2)	2 (4.4)	3 (6.7)	2 (4.4)
Once every 2-4 years	4 (8.9)	4 (8.9)	2 (4.4)	3 (6.7)	4 (8.9)	3 (6.7)	6 (13.3)	3 (6.7)
Once every 5+ years	2 (4.4)	2 (4.4)	1 (2.2)	2 (4.4)	3 (6.7)	2 (4.4)	1 (2.2)	1 (2.2)
Other frequency[1]	3 (6.7)	6 (13.3)	1 (2.2)	3 (6.7)	6 (13.3)	5 (11.1)	5 (11.1)	3 (6.7)

[1]Other frequency: 1-3 x within 2 weeks every 3 months; 1-2 times per study; depends on study, as required. Other populations (< once a year): 0-24 months; adults > 15 years; children 6-23.9 months; households; Participants could provide multiple responses to this question.

Supplementary Table S3: Which of the following mode(s) do you use for collection of individual dietary intake data?

**Mode for collecting intake data**	**Self-administered by person whose diet is being measured**		**Interviewer-administered by research assistant/field worker**		**Direct observation by research assistant/field worker**		**Passive collection (wearable device and/or camera)**		**Other method[1]**	
	n	%	n	%	n	%	n	%	n	%
24-hr recall (24R)	7	15.6%	37	82.2%	6	13.3%	1	2.2%	1	2.2%
Food Frequency Quest. (FFQ)	8	17.8%	25	55.6%	6	13.3%	2	4.4%	1	2.2%
Weighed food record (WFR)	3	6.7%	8	17.8%	4	8.9%	1	2.2%	1	2.2%
Estimated food record (estFR)	5	11.1%	6	13.3%	3	6.7%	2	4.4%	1	2.2%
Image-based food record (IBFR)	1	2.2%	7	15.6%	2	4.4%	1	2.2%	0	0.0%
Diet history (DHx)	5	11.1%	11	24.4%	4	8.9%	2	4.4%	1	2.2%
Image-assisted method (IAM)	2	4.4%	7	15.6%	0	0.0%	1	2.2%	0	0.0%
Other[2]:	1	2.2%	6	13.3%	0	0.0%	0	0.0%	0	0.0%

[1]Other administered methods were not specified. [2]Other DA methods: 7-day FFQ of targeted items; Diet quality tool; Dietary Diversity Score for Women; Food Atlas; Verbal interview format; Dietary Diversity Score for IYCF; duplicate method. Participants could provide multiple responses to this question.

Supplementary Table S4: Which of the following approaches and tools do you use during the collection of intake data in the field?

Approaches and tools for collecting intake data	Pen & paper records		Generic digital form via survey platform (eg, Kobo, ODK, Qualtrics)		Standalone mobile app developed for dietary intake assessment (eg, app for collecting a recall or images)		Wearable device (eg, camera)		Fixed device (eg, mounted camera)		Other applications or methods	
	n	%	n	%	n	%	n	%	n	%	n	%
24-hr recall (24R)	31	68.9%	20	44.4%	3	6.7%	1	2.2%	0	0.0%	2	4.4%[1]
Food frequency Quest. (FFQ)	22	48.9%	14	31.1%	4	8.9%	0	0.0%	0	0.0%	0	0.0%
Weighed food record (WFR)	8	17.8%	5	11.1%	3	6.7%	0	0.0%	0	0.0%	0	0.0%
Estimated food record (estFR)	7	15.6%	6	13.3%	3	6.7%	0	0.0%	0	0.0%	0	0.0%
Image-based food record (IBFR)	3	6.7%	1	2.2%	4	8.9%	1	2.2%	1	2.2%	0	0.0%
Diet history (DHx)	11	24.4%	7	15.6%	3	6.7%	0	0.0%	0	0.0%	0	0.0%
Image-assisted method (IAM)	7	15.6%	2	4.4%	2	4.4%	1	2.2%	0	0.0%	0	0.0%
Other[2]:	0	0.0%	5	11.1%	1	2.2%	0	0.0%	0	0.0%	1	2.2%

[1]Other approaches reported by participants to be used with 24R: Real-size picture booklet of foods and drinks; Food models
[2]Other DA methods: 7-day FFQ of targeted items; Diet quality tool; Dietary Diversity Score for Women; Food Atlas; Verbal interview format; Dietary Diversity Score for IYCF; duplicate method. Participants could provide multiple responses to this question.

Supplementary Table S5: For the dietary assessment method(s) you indicated that you currently use, please provide some more information about the approach(es) you use to analyse (or code) the collected individual dietary intake data to derive estimates of nutrient and/or food intake, diet quality, dietary diversity etc.

Approaches to analysis of collected intake data, by DA method	Manual analysis by a person using a non-specific dietary assessment software (e.g. Microsoft Access, Microsoft Excel)		Manual analysis by a person using standalone nutrition assessment software (e.g. Nutrition Data System for Research, Nutritics) or purpose-built software.		Semi-automated analysis where the person completing manual analysis is supported by the automation of one or more tasks (e.g. calculation of nutrient intake occurs automatically after manual entry of intake data)		Fully automated analysis without any person involvement (e.g. image-based food record data where the identification and quantification of data collected is performed automatically using computer vision algorithms)		Other analysis approach[1]	
	n	%	n	%	n	%	n	%	n	%
24-hr recall (24R)	18	40.0%	16	35.6%	13	28.9%	5	11.1%	2	0.2%
Food frequency Quest. (FFQ)	14	31.1%	10	22.2%	11	24.4%	1	2.2%	1	0.0%
Weighed food record (WFR)	6	13.3%	4	8.9%	7	15.6%	2	4.4%	0	0.1%
Estimated food record (estFR)	5	11.1%	4	8.9%	4	8.9%	1	2.2%	0	0.0%
Image-based food record (IBFR)	3	6.7%	3	6.7%	3	6.7%	2	4.4%	0	0.1%
Diet history (DHx)	5	11.1%	7	15.6%	6	13.3%	0	0.0%	0	0.0%
Image-assisted method (IAM)	4	8.9%	1	2.2%	3	6.7%	1	2.2%	0	0.0%
Other[2]:	6	13.3%	3	6.7%	1	2.2%	0	0.0%	0	0.0%

[1]Responses by participants of analysis approaches to be used with 24R were SPSS and FAO individual dietary assessment method; and with FFQ was PSS (not further defined by participants). [2]Other DA methods: 7-day FFQ of targeted items; Diet quality tool; Dietary Diversity Score for Women; Food Atlas; Verbal interview format; Dietary Diversity Score for IYCF; duplicate method. Participants could provide multiple responses to this question.

Supplementary Table S6: For the dietary assessment method(s) you indicated that you currently use, please provide some information about how you interpret the analysed individual dietary intake data through comparisons to recommendations (eg, nutrient intake recommendations, food guidance systems).

Approach to interpretation of analysed dietary intake data	Manual only with no automation		Semi-automated where the person(s) performing the interpretation is supported by the automation of one or more tasks (e.g. calculation of %RDI)		Fully automated analysis where the interpretation occurs without any person(s) involvement		Other interpretation approach[1]	
	n	%	n	%	n	%	n	%
24-hr recall (24R)	17	37.8%	23	51.1%	3	6.7%	0	0.0%
Food frequency Quest. (FFQ)	11	24.4%	19	42.2%	3	6.7%	2	4.4%
Weighed food record (WFR)	5	11.1%	6	13.3%	2	4.4%	0	0.0%
Estimated food record (estFR)	5	11.1%	4	8.9%	1	2.2%	0	0.0%
Image-based food record (IBFR)	4	8.9%	4	8.9%	0	0.0%	0	0.0%
Diet history (DHx)	5	11.1%	10	22.2%	0	0.0%	0	0.0%
Image-assisted method (IAM)	2	4.4%	6	13.3%	0	0.0%	0	0.0%
Other[2]:	4	8.9%	3	6.7%	0	0.0%	0	0.0%

[1]Other approaches used with FFQ: Frequencies and percentages; no specific comparison to recommendations; [2]Other DA methods: 7-day FFQ of targeted items; Diet quality tool; Dietary Diversity Score for Women; Food Atlas; Verbal interview format; Dietary Diversity Score for IYCF; duplicate method. Participants could provide multiple responses to this question.
